# A comparison of continuous and discrete time modeling of affective processes in terms of predictive accuracy

**DOI:** 10.1038/s41598-021-85320-4

**Published:** 2021-03-18

**Authors:** Tim Loossens, Francis Tuerlinckx, Stijn Verdonck

**Affiliations:** KU Leuven, University of Leuven, Leuven, Belgium

**Keywords:** Computational science, Statistics, Psychology

## Abstract

Intra-individual processes are thought to continuously unfold across time. For equally spaced time intervals, the discrete-time lag-1 vector autoregressive (VAR(1)) model and the continuous-time Ornstein–Uhlenbeck (OU) model are equivalent. It is expected that by taking into account the unequal spacings of the time intervals in real data between observations will lead to an advantage for the OU in terms of predictive accuracy. In this paper, this is claim is being investigated by comparing the predictive accuracy of the OU model to that of the VAR(1) model on typical ESM data obtained in the context of affect research. It is shown that the VAR(1) model outperforms the OU model for the majority of the time series, even though time intervals in the data are unequally spaced. Accounting for measurement error does not change the result. Deleting large abrupt changes on short time intervals (that may be caused by externally driven events) does however lead to a significant improvement for the OU model. This suggests that processes in psychology may be continuously evolving, but that there are factors, like external events, which can disrupt the continuous flow.

## Introduction

Many processes in psychology are conceived as continuously unfolding across time. A prime example is the study of the dynamics of affect^1,2^. To study the intra-individual dynamics (e.g., of affect), researchers often rely on intensive longitudinal studies. Because of its high ecological validity, a specific method that is frequently used to capture people’s everyday experiences is the experience sampling method (ESM)^3,4^. Such intensive longitudinal studies result in time series data at the level of the individual. A time series consist of several measurements of different variables and the exact time stamps of when the measurements were obtained. They can be used to study the (lagged) relationships between some variables of interest or the self-correlation of a variable across time^5^.

To analyze such multivariate time series, many modeling approaches exist. The goal of the modeling approaches is to formalize the laws which govern the evolution of the observed processes in a mathematical framework. These approaches can be partitioned into two major classes depending on the treatment of time. There are discrete-time and continuous-time approaches^5–10^.The class of discrete-time modeling approaches encompasses the autoregressive (AR) models, often referred to as vector autoregressive (VAR) models when multiple variables are at play^5–7,11^. Such models are characterized by recursive relations or difference equations. Continuous-time models, like the Ornstein–Uhlenbeck (OU) model (alternatively referred to as the continuous-time VAR(1) model^7^), rely on differential equations instead^7,9,10,12^.

In many ESM studies, the time intervals are of unequal length^7^. Although one could design studies with measurements that are equally spaced in time, unpredictable measurements are beneficial for the ecological validity of ESM data^4^. Moreover, unequally spaced measurements can be more efficient for gathering information about the underlying process^10^. A primary difference between discrete-time and continuous-time models is that the latter take into account the exact time interval between measurements while the former do not—discrete-time models assume equally spaced time-intervals. Also, continuous-time models fit more naturally with the idea that many intra-individual processes in psychology are continuously evolving across time—the processes continue to exist and evolve in between measurements, and therefore continuous-time models are a more natural choice.

The choice for discrete-time models is often driven by pragmatic reasons because they are simpler to formulate, fit and understand. However, not taking into account unequal time intervals can lead to biased model estimates^7^, which has ramifications for interpretation and could lead to false conclusions regarding the interplay of specific processes within an individual as well as how the interplay manifests itself for different individuals. The demonstration of such issues largely relies on simulation-based techniques where data are simulated from a continuous time model (the OU model) using unequally spaced sampling schemes. The severity of bias depends on sample size and autocorrelation.

Hence, both common sense and the simulation studies tell us that if the underlying process is continuously evolving and the time intervals between measurements are unequal, it is favorable to use a continuous-time model—it is to be expected that such a continuous-time model would be a better description of the data than its discrete-time counterpart. A direct consequence should be that we can make more accurate predictions of future data (i.e., forecasting) using a continuous-time framework. Especially since time intervals are important both for obtaining good estimates and for accurately predicting the state of the system—how much the system can have changed depends on how long we wait to observe it again.

All these considerations sound perfectly reasonable. However, to the best of our knowledge, this issue has not yet been addressed. Is it truly the case that, for example, ESM data of affect dynamics with unequal time intervals are better modeled with continuous-time models than with discrete-time models? To investigate this question, we will compare the predictive accuracy of the continuous-time Ornstein–Uhlenbeck model to that of the discrete-time VAR(1) model using ESM data from two large studies obtained in the context of affect research. A first possibility is that the predictive accuracy of the Ornstein–Uhlenbeck model will be better than that of the VAR(1) model because the time intervals in the data are unequally spaced. However, there is also a second possibility. As we will explain in the section on “How VAR and OU deal with time”, it is also possible that the OU is a worse model for actual data.

## Background on the statistical models

### The VAR(1) model

When participants in an ESM study have, for example, rated the intensity of their affect at each measurement occasion, or have reported on specific emotions, behaviors or thought processes, several (multivariate) time series of measurements are obtained (one time series per participant). Let ${\mathbf {x}}_{pi} = \{x_{1i}, \ldots , x_{di} \}_{p}$ denote the set of *d* different ratings (the variables) from a specific participant *p* at measurement occasion $i = 0, \ldots , N$. For simplicity, the index *p* referring to the specific participant will be ignored.

The VAR(1) model describes a linear dependency between consecutive measurements and is given by

1 $$\begin{aligned} {\mathbf {x}}_{i} = {\mathbf {c}} + A {\mathbf {x}}_{i-1} + \varvec{\epsilon }_{i}. \end{aligned}$$

The parameter ${\varvec{c}} \in {\mathbb {R}}^{d}$ denotes the intercept, $A \in {\mathbb {R}}^{d \times d}$ is the transition matrix, and $\varvec{\epsilon }_{i}$ is a vector containing the stochastic fluctuations (innovations) at measurement occasion *i*.

The innovations $\varvec{\epsilon }_{i}$ are assumed to be Gaussian distributed with mean zero and covariance $\Sigma _{\varvec{\epsilon }}$. They are assumed to be uncorrelated across time. The system of Eq. (1) is therefore nothing but a system of multivariate linear regression equations, modeling each variable as a linear function of the variables at the previous measurement.

The diagonal elements of the transition matrix *A* represent the autoregressive parameters of the system, while the off-diagonal elements correspond to the cross-lagged effects between the variables. The eigenvalues of *A* determine whether the VAR(1) process is stable or not. If all the eigenvalues have a modulus strictly smaller than one, the first moment, second moment and the autocovariance of the unconditional (joint) distribution of the measurements ${\mathbf {x}}_{i}$ are time invariant. In that case, provided with enough data, the measurements ${\mathbf {x}}_{i}$ are Gaussian distributed with mean

2 $$\begin{aligned} \varvec{\mu } = (I_{d}-A)^{-1}{\mathbf {c}}, \end{aligned}$$

and covariance $\Sigma$. Here, $I_{d}$ denotes the *d*-dimensional identity matrix. This covariance is related to that of the innovations as

3 $$\begin{aligned} \Sigma _{\varvec{\epsilon }} = \Sigma - A \Sigma A^{T}. \end{aligned}$$

If some of the eigenvalues of *A* fall on the unit circle, we have an unstable VAR(1) process whose variance (and covariance) will grow infinitely large. Furthermore, if at least one of the eigenvalues of *A* has a modulus strictly larger than 1, the expectation value itself will drift towards (minus) infinity as time goes on. In practice, the measurement scales that are used to collect answers on affect questions are bounded. Therefore, divergent patterns will never occur. The VAR(1) model is generally limited to only include those models that are stable (and thus stationary). We will do so as well.

Returning to the stable case, the Gaussian distribution composed of the mean $\varvec{\mu }$ and the covariance $\Sigma$ is independent of time—it is stationary. It tells us what the distribution of the observations ${\mathbf {x}}_{i}$ would be, if we were to observe the system for an infinitely long time. If we have no prior information regarding the previous state of the system at measurement occasion *i*, our best guess would be that the observation ${\mathbf {x}}_{i+1}$ falls somewhere within this stationary Gaussian distribution. However, if we do know ${\mathbf {x}}_{i}$, then we can make a more accurate prediction using the expectation value

4 $$\begin{aligned} \langle {\mathbf {x}}_{i+1} \rangle = {\mathbf {c}} + A {\mathbf {x}}_{i} \end{aligned}$$

and the covariance $\Sigma _{\varvec{\epsilon }}$ of the innovations.

### The Ornstein–Uhlenbeck model

An OU process is a continuous-time stochastic process that results in a VAR(1) process when the observations are equally spaced in time. Assume there are *d* different continuous-time processes ${\mathbf {y}}(t) = \{ y_{1}(t), \ldots , y_{d}(t) \}$ underlying the measurements ${\mathbf {x}}_{i}$, $i = 0, \ldots , N$, so that

5 $$\begin{aligned} {\mathbf {x}}_{i} = {\mathbf {y}}(t_{i}) \end{aligned}$$

where $t_{i}$ denotes the time at measurement occasion *i*. The process ${\mathbf {y}}(t)$ constitutes an OU process if its evolution is governed by the stochastic differential equation

6 $$\begin{aligned} \mathrm {d}{\mathbf {y}}(t) = \theta (\varvec{\mu }-{\mathbf {y}}(t)) \mathrm {d}t + \sigma \mathrm {d}{\mathbf {W}}(t). \end{aligned}$$

The matrix $\theta \in {\mathbb {R}}^{d\times d}$ contains the autoregressive and cross-regressive effect, $\varvec{\mu } \in {\mathbb {R}}^{d}$ denotes the center and corresponds to the stationary mean of the process ${\mathbf {y}}(t)$ (provided it is stable), and ${\mathbf {W}}(t)$ is a vector containing *d* distinct Wiener processes whose covariance is given by $\sigma \sigma ^{T}$. Without loss of generality, the matrix $\sigma \in {\mathbb {R}}^{d\times d}$ can be assumed to be a triangular matrix. The process is stable if the real parts of the eigenvalues of $\theta$ are strictly larger than zero.

Equation (6) governs the evolution of ${\mathbf {y}}$ over an infinitesimally short period of time. Due to the Wiener processes, this evolution is inherently stochastic. Integrating the small changes in ${\mathbf {y}}$ over time, the state of the system can be expressed in function of a previous state ${\mathbf {y}}(t_{i-1})$ at time $t_{i-1}$ and the elapsed time interval $\Delta t_{i} = t_{i} - t_{i-1}$^13^. For the integral formulation of the OU model, see (A.1) in Appendix A. From the integral form of the OU model, we can see that if the time intervals are all equal, $\Delta t_{1} = \Delta t_{2} = \cdots = \Delta t$, then the OU model becomes equivalent with a VAR(1) model; the parameters $\varvec{\mu }$ and $\theta$ are then related to the intercept ${\mathbf {c}}$ and the transition matrix *A* of the VAR(1) model as follows (see Appendix A)

7 $$\begin{aligned} {\mathbf {c}}&= \big ( I_{d} - e^{-\theta \Delta t} \big ) \varvec{\mu } \end{aligned}$$

8 $$\begin{aligned} A&= e^{-\theta \Delta t} . \end{aligned}$$

When the measurements are unequally spaced in time, the general solution of the expectation value of the OU model is given by^13^

9 $$\begin{aligned} \langle {\mathbf {y}}(t_{i+1}) \rangle = \big ( I_{d} - e^{-\theta \Delta t_{i}} \big ) \varvec{\mu } + e^{-\theta \Delta t_{i}} {\mathbf {y}}(t_{i-1}) \end{aligned}$$

and the (conditional) covariance is given by

10 $$\begin{aligned} \Sigma _{\Delta t_{i+1}} = \Sigma _{{\mathbf {y}}} - e^{-\theta \Delta t_{i+1}} \Sigma _{{\mathbf {y}}} e^{-\theta ^{T}\Delta t_{i+1}}. \end{aligned}$$

When prior information is known about a previous observation, the expectation value (9) and the covariance (10) can be used for predictions.

### How VAR and OU deal with time

The OU model describes a system ${\mathbf {y}}(t)$ that is continuously changing across time. We will therefore have different expectations regarding its state depending on the length of the time interval between the last observation and now. This is made explicit in the expressions for the conditional mean (9) and the conditional covariance (10) which both explicitly depend on the time interval. According to both the VAR(1) and the OU model, the state ${\mathbf {y}}$ is expected to relax towards the stationary mean $\varvec{\mu }$ after a measurement (assuming no disruptions occurred), because this is the most probable region for the state to be if we wait for a very long time before looking at the system again. Likewise, the conditional covariance which reflects the uncertainty we have concerning the state evolves towards the stationary covariance $\Sigma _{{\mathbf {y}}}$; the longer we wait, the less information is gained from knowing the previous state. From Eqs. (9–10) we see that, for the OU model, this relaxation depends exponentially on the time interval $\Delta t$ and the system interactions $\theta$. The relaxation time is determined by the transition matrix $\theta$; more specifically, the real parts of the eigenvalues of $\theta$. Larger real parts imply a faster relaxation and thus a shorter relaxation time (the time it takes to reach equilibrium is shorter). For the VAR(1) model, by virtue of the relation (8), this means that the autocorrelation of the process is determined by the transition matrix *A*, which includes both the interactions of the system and the time scale on which the system is observed.

Because the dynamics described by the VAR(1) model is so similar to the dynamics described by the OU model, we can identify regimes in which the models become indistinguishable. First, if the time intervals between the measurements are equally spaced, there is a one-to-one correspondence between the VAR(1) model and the OU model. In that case, the parameter estimates of the VAR(1) model can be mapped onto the parameter estimates of the OU model and they will have the same predictive accuracy. Second, when the time intervals between the measurements are long in comparison to the relaxation time of the system, then the expectation value (9) of the OU model will have relaxed towards the mean $\varvec{\mu }$ and the conditional covariance (10) will have relaxed to the stationary covariance $\Sigma _{{\varvec{y}}}$. For the VAR(1) model this corresponds to a situation for which Eqs. (2) and (4) reduce to $\langle {\mathbf {x}}_{i+1} \rangle \approx {\mathbf {c}} \approx \varvec{\mu }$, and Eq. (3) reduces to $\Sigma _{\varvec{\epsilon }} = \Sigma$. In such a situation, the data are best described by a stationary Gaussian distribution with mean $\varvec{\mu }$ and covariance matrix $\Sigma$ (note that $\Sigma = \Sigma _{{\mathbf {y}}}$), ignoring the dynamics contained in the transition matrices $\theta$ and *A*. Since the OU model and the VAR(1) model will have the same stationary distribution in this scenario, their predictive accuracy should be comparable (there can always be small differences because they deal differently with the time intervals). In practice, however, autocorrelations are generally large enough to detect dynamical relaxation patterns.

It is only when the measurements are unequally spaced and the time scale of the measurements smaller than the relaxation time of the system that differences between the models start to appear. The OU model explicitly specifies that how much the system can have changed and how large our uncertainty is depends on the time interval (see Eqs. (9) and (10)). The VAR(1) model does not have this specificity and makes the same prediction regardless of the time interval. This could lead to false conclusions; if the underlying process is truly an Ornstein–Uhlenbeck process, the VAR(1) model will overestimate how much the system can have changed on smaller time intervals and underestimate the change on longer time intervals. Because the VAR(1) model does not account for the time interval in the prediction, we expect it to have a worse predictive accuracy than the OU model, provided that the underlying process is a continuous OU process.

However, things might be different when OU is a misspecified model and the dynamics of the process under consideration do not coincide with those of an OU process (assuming the evolution of the process is continuous in time). In such situation, being too specific about the time evolution might in fact become harmful for predictions. For an OU process, we expect to see a specific kind of exponential relaxation, both in the expected value and the variance-covariance on different time scales. If the dynamical patterns in the data diverge from the expected pattern, the Ornstein–Uhlenbeck process will also have to make compromises to describe the observed patterns to the best of its capabilities. This may cause the OU model to make bad predictions.

A divergent dynamical pattern that can lead to an underestimation of the relaxation time (i.e., the relaxation time of the estimated process is a lot shorter than the true relaxation time) is the occasional occurrence of large changes on small time intervals. In order to accommodate large changes on small time scales in a continuous manner, the system has to evolve more rapidly. Therefore, the system will relax more rapidly, or equivalently, the relaxation time will become much shorter. In extreme cases, such abrupt changes could require the OU model to completely relax on this short time interval and ignore the relaxation patterns that it would otherwise capture nicely. In such an extreme case, an simple stationary Gaussian distribution may also outperform the OU model (and VAR model) in terms of predictive accuracy, because it is a simpler model. Large changes can also pose a problem for the discrete-time VAR(1) model. However, the effect of large changes on small time intervals are typically diminished when the time intervals are ignored and the spread of intervals is large.

There are many ways in which misspecification of the OU model can happen. We will focus on two possible scenarios that may lead to worse predictions compared to the VAR(1) model. In the next paragraphs, we discuss likely scenarios in which relaxation dynamics are being distorted.

**Figure 1:**
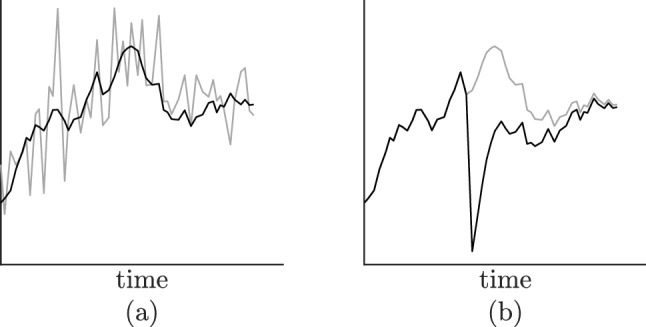
(**a**) The dark line is an illustration of a one-dimensional OU process. The lighter line corresponds to the same OU process, but with additional measurement error. (**b**) An OU process that has been disrupted by an external event (the larger spike in the middle). For reference purposes, the trajectory that would be observed if there was no disruption is depicted as well (light).

A first scenario is the presence of measurement error causing large deviations. Typical for measurement error is that it does not influence the dynamical process itself, only the measurementss. In such a case, the observations we obtain do not truly coincide with the true state of the system at the moment of measurement, but contain some additional noise. This means that the observed fluctuations are actually larger than the dynamic fluctuations of the system. Hence, the dynamical pattern becomes obscured and it becomes more difficult to accurately determine the interactions $\theta$ and the natural system fluctuations described by the covariance $\sigma \sigma ^{T}$. An illustration is given in Fig. 1a.

A second scenario is the influence of external events, which may also augment the observed fluctuations. Contrary to measurement noise, however, the system is truly influenced by these external vagaries and can be pushed into configurations that would otherwise be impossible to attain. If a student receives exam results that are unexpectedly positive, she might experiences levels of euphoria and relief beyond normal, daily limits. Depending on the relaxation time of her affect system, these levels may remain above the normal limits for longer periods of time. This is not the case with measurement noise: if we observe a state beyond normal limits, this does not propagate to ensuing observations. Hence, for external events, the post-event dynamics could behave OU-like, but the event-driven jumps cannot be explained by an OU model. An illustration is given in Fig. 1b. The estimated OU model is likely to have a shorter relaxation time than the true underlying process in order to accommodate the event-driven jump.

In this paper, starting from the idea that the measured process (in the case of this paper, the process is affect) is a continuously evolving process, we will compare the predictive accuracy of the continuous-time OU model with that of the (popular) discrete-time VAR model. The primary aim of using cross-validation as an analysis tool is model selection—which model in general has the best description of the data (model complexity taken into account)? As stated by Breiman (2001)^14^: “The most obvious way to see how the model box emulates nature’s box is this:... fit the parameters in your model by using the data, then, using the model, predict the data and see how good the prediction is.” Besides comparing the predictive accuracy of the basic OU model with that of the basic VAR model, we will also extend both models to account for measurement noise. This extension is done by recasting the OU model and the VAR model as state space models. Additionally, we will examine what happens to the predictive accuracy of the models when large changes on small time scales are ignored (i.e. they are assumed to be generated by a different model than the one being studied and are not taken into account for estimating the model parameters). Since we believe that large deviations on small time scales have a larger impact on the OU model than on the VAR model, we foresee that the OU model’s predictive accuracy will benefit more from removing extreme observations.

## Materials and methods

To investigate how the predictive accuracy is influenced when the exact time intervals between measurements are taken into account, we did walk-forward cross validation on 685 affect time series from two different ESM studies. We did not collect the data ourselves. A time series is composed of ratings on several affect items which were reported at every measurement occasion. We analyzed both the raw multivariate time series and the two-dimensional time series obtained by averaging the scores of the positive and negative items respectively. These momentary averages are referred to as positive (PA) and negative affect (NA). In both cases, more or less the same results were obtained. Hence, we only report the results pertaining to the two-dimensional data. In this section, we give an overview of the data and we discuss the different analyses that we did.

### Data

#### Study 1: three-wave ESM study

This study concerned the assessment of changes in the affect dynamics of freshmen students going through their first year of college and consisted of three separate assessment periods or measurement waves^1^. It was approved by the institutional review board of the KU Leuven (ML8514-S54567) and complied with local ethical regulations. All participants gave informed consent^1^.

Each assessment period was comprised of a week, during which the students’ momentary affect was tracked using a typical ESM protocol. The first ESM assessment was done at the beginning of the academic year. The second and third assessments followed respectively four and twelve months later. During the recruitment stage, special attention was paid to recruiting a sample of students that reflected a broad range of psychological well-being in terms of severity of depressive symptoms. The students were remunerated for their participation; they earned €60 for each wave completed and another €60 for completing all waves. Initially, 180 participants were selected from a pool of 686 students (235 males) starting freshman year. To ensure the desired sample size, however, an additional 22 participants were recruited after the study had already begun. This led to an initial sample of 202 participants. Because of a remarkably low compliance, two students were excluded from the study (they had a compliance below 50% in the first wave). As such, the final study was involved 200 freshmen students (55% female; $M_{\text {age}} = 18$ years; $SD_{\text {age}} = 1$ year). Due to dropout, 190 participants (56% female) remained in Wave 2, and 177 participants (56% female) remained in Wave 3^1^.

For the purposes of this paper, time series from different waves but from the same participant were treated separately, resulting in a total of 567 time series for this study.

**The ESM protocol** Each measurement wave, the participants’ momentary affect was tracked using ESM. A Motorola Defy Plus Smartphone was given to each of the participants. The participants had to carry around this device as they went through their daily activities. The smartphones were programmed to beep 10 times a day in between 10 am and 10 pm for seven consecutive days. The beep times were randomly sampled using a stratified random interval scheme. Every 72 minutes, on average, a beep prompted the participants to rate their positive (*happy*, *relaxed*, *cheerful*) and negative (*sad*, *anxious*, *depressed*, *anger*, *stressed*) emotions by means of a continuous slider ranging from 0 (*not at all*) to 100 (*very much*). On average, participants responded to 87% ($SD = 9\%$), 88% ($SD = 9\%$) and 88% ($SD = 9\%$) of the beeps for the three waves respectively^1^.

**PA and NA scores** For each beep, positive affect (PA) and negative affect (NA) scores were constructed by averaging the responses to the individual positive and negative emotion items respectively.

#### Study 2: clinical ESM study

This study was designed to study symptom and emotion dynamics in individuals suffering from major depressive disorder and/or borderline personality disorder^15^. It complied with local ethical regulations and was approved by the Medical Ethics Committee UZ Leuven (B322201627414). All participants provided informed consent^15^.

To recruit participants, a clinician screened patients in thee Belgian psychiatric wards (*KU Leuven hospital UPC Sint-Anna*; *UPC De Weg/Onderweg*; and *Broeders Alexianen Tienen hospital ward Prisma II*). A clinically trained researcher subsequently interviewed those patients who were deemed eligible for enrollment. For the interview, the Dutch version of the Structured Clinical Interview for DSM axis I disorders (SCID-I^16^) and the Borderline Personality Disorder (BPD) subscale of the DSM axis II disorders (SCID-II^17^) were used. If patients met the criteria for one of the mood or personality disorders, they were included, unless they were acutely psychotic, acutely manic, addicted, or diagnosed with a (neuro)cognitive disorder at the time of the interview^15^.

Besides recruiting patients from psychiatric wards, a sample of healthy controls was recruited via advertisements, social media, flyers and by the Experimental Management System of the KU Leuven (university of Leuven). The age and gender distribution of the healthy controls were made to match those of the clinical patients. Some participants were removed of the initial sample (clinical patients as well as healthy controls) because of complications during the ESM protocol or because of a low compliance. The final sample was comprised of 118 participants of whom 38 were diagnosed with major depressive disorder (50% female; $M_{\text {age}} = 41$ years; $SD_{\text {age}} = 14$ years), 20 with borderline personality disorder (95% female; $M_{\text {age}} = 30$ years; $SD_{\text {age}} = 12$ years), another 20 with both (85% female; $M_{\text {age}} = 33$ years; $SD_{\text {age}} = 11$ years), and a control group without current psychiatric diagnosis consisting of 40 participants (58% female; $M_{\text {age}} = 35$ years; $SD_{\text {age}} = 12$ years)^15^.

Each of the recruited participants had to partake in a one week ESM protocol. A compensation was given to the participants after the ESM assessment. Those who had a compliance rate of at least 75% received €35. Five euros were deducted for every ten percent that the compliance rate was below this threshold^15^.

**The ESM protocol** The measurement protocol was similar to the one of study 1. The participants were given a Motorola Defy Plus Smartphone which they had to carry around as they went trough their daily activities. The devices were programmed to beep 10 times a day in between 10 am and 10 pm for seven consecutive days. A stratified random interval scheme was used to sample the beep times; a beep occurred on average every 72 minutes. Each beep prompted the participants to fill out a questionnaire which consisted of 27 questions, including questions about emotions, social expectancy, emotion regulation, context and psychiatric symptoms. On average, it took participants 2’2” ($SD = 37''$) to fill out a questionnaire. The positive (*euphoric*, *happy*, *relaxed*) and negative (*depressed*, *stressed*, *anxious*, *angry*) emotion items could be rated using a continuous slider ranging from 0 (*not at all*) to 100 (*very much*). On average, the participants responded to 87% ($SD = 11\%$) of the beeps^15^.

**PA and NA scores** As for Study 1, the PA and NA scale scores were calculated as average item responses.

## Methods

The two central models in this paper are the discrete-time VAR(1) model and its continuous-time counterpart, the OU model. The basic model formulations do not include measurement error. For both models, we formulated a variant with measurement error. We also included a stationary Gaussian distribution as a model to deal with time series in which there is no significant autocorrelation, or time series for which divergent dynamical patterns lead to an underestimation of the relaxation time.

After fitting all these models to the data, their predictive accuracy was evaluated by means of out-of-sample cross-validation^18^. Because it was hypothesized that large changes in the affect state on short time spans are more difficult for a continuous-time model to deal with, the predictive accuracies of the VAR(1) and OU model, without and with measurement error, were also evaluated after removing extreme observations.

### Model fitting

#### Models without measurement error

To fit the models, we relied on maximum-likelihood optimization. Given the measurement ${\mathbf {x}}_{i}$ at time $t_{i}$ and the set of parameters $\{ \varvec{\mu },\theta ,\sigma \}$, the OU model predicts that the ensuing measurement ${\mathbf {x}}_{i+1}$ at time $t_{i+1}$ falls somewhere in the Gaussian distribution with mean (9), where ${\mathbf {y}}(t_{i}) = {\mathbf {x}}_{i}$, and conditional covariance (10). The likelihood of the data point ${\mathbf {x}}_{i+1}$ is found by inserting it into the expression of this Gaussian distribution. The min-log-likelihood is the negative logarithm of this value. The min-log-likelihood of the entire time series given a parameter set is simply the sum of all the min-log-likelihoods of the individual data points. Hence, given a training set, the min-log-likelihood is a function of the model parameters only. The parameter estimates were found by minimizing this function with respect to the model parameters.

To minimize the min-log-likelihood function, we relied on the differential evolution heuristic^19^, which is a global optimizer. For each optimization, we considered $NP = 50$ agents. Furthermore, we used the binomial DE/rand/1 crossover strategy with a probability rate $CR = 0.6$. The number of iterations that was considered is 3000. For the weighing factor *F* we use a uniform random distribution *U*(0, 2)^20^.

In order to find the estimates of the VAR(1) model, we relied on the same procedure as for the OU model, but we set the time intervals $\Delta t_{i}$ equal to 1. The VAR(1) estimates were then found using the equivalence (A.2–A.4).

To evaluate our estimation procedure, we studied the analytical, closed-form estimates of the VAR(1) model with those obtained from the likelihood procedure, for those cases where the closed-form estimates resulted in a stable transition matrix (in our estimation procedure, this constraint is imposed, but this constraint is not imposed on the closed-form solutions). The results of this study can be found in Appendix B.

#### Models with measurement error

 To include measurement noise, we recast the OU model and the VAR model as state-space models. The state-space models include two extra parameters compared to their basic forms: the variance of the measurement error in the PA dimension and the variance of the measurement error in the NA dimension. The models with measurement error were fitted using the marginal likelihood procedure (based on the linear Kalman filter approach^21^). The differential evolution heuristic was used to minimize the marginal min-log-likelihood. The same marginal likelihood was used in the out-of-sample cross-validation to compute the predictive accuracy.

### Predictive accuracy: out-of-sample cross-validation

To compare the different models, we evaluated how well they predict previously unseen data (data not used for fitting the model). We used a walk-forward procedure. As in all cross-validation methods, the time series is split into two parts: a training set and a test set. Specifically to the walk-forward method, the training sets consists of the first $N-n$ data points. It is used to fit the model (i.e., to find parameter estimates for the model of interest). The parameter estimates are then used to predict the measurement following the last of the training set (i.e. the $(N-n+1)$th measurement). The measurement that is being predicted makes up the test set. The likelihood (or min-log-likelihood) of the test data point given the prediction is an indicator for the model’s predictive accuracy. To get a more robust estimate of the predictive accuracy, it is common practice to vary the sample size $N-n$ of the training set and average the predicted min-log-likelihoods. Note that the information content of the training set reduces as *n* increases, which in general increases the uncertainty of predictions. In this paper, the *n* was varied from 1 to 10 for every time series, resulting in 10 predicted min-log-likelihoods per time series which were then averaged.

### Removal of large deviations

In order to identify large deviations, we define a speed measure. For two consecutive measurements ${\mathbf {x}}_{i}$ and ${\mathbf {x}}_{i+1}$, we defined the speed $v_{i+1}$ as

$$\begin{aligned} v_{i+1} = \frac{1}{\Delta t_{i+1}} \sqrt{\sum _{j = 1}^{d} \left( \frac{x_{j,i+1} -x_{ji}}{s_{j}} \right) ^{2} }, \end{aligned}$$

where $s_{j}$ denotes the sample standard deviation of the observations $x_{j}$. This speed is equal to the Euclidean displacement of the normalized ratings divided by the time interval which separates them. This speed is influenced by both the magnitude of the displacement and the length of the time interval and will become especially large for large changes on small time scales. We normalize the ratings to ensure that speed is invariant to the scaling of the dimensions (and thus not dominated by those dimensions with larger variability or larger measurement scales).

The median speed for a person is calculated and next, the median absolute deviations (MAD). Measurements above some MAD cutoff (denoted as *C*) were ignored in the training sets and the models were fitted to the truncated data (i.e., transitions involving this large change were not considered, but the end point was still used as a starting point to compute the likelihood of the ensuing observation). Then, the predictive accuraciy was computed using the out-of-sample cross-validation described before. This analysis was repeated for a range of thresholds (varying *C* from 10 down to 1). The smaller *C*, the more speeds are removed. An analysis with all data included has $C=\infty$.

#### Missing observations and nights

The time series that we consider were collected over a seven day period. Several measurements were collected during each day in between 10 am and 10 pm. As a consequence, the time interval between the last measurement of a day and the first measurement of the next is long in comparison to the time interval in between two daytime measurements. Because of these long time intervals, the OU model will almost entirely relax towards its stationary distribution before the first measurement in the morning. If we, however, consider the night as a single transition for the VAR(1) model, it can never reach stationarity. This may introduce a bias in favor of the OU model. Hence, instead, we assume the first measurement of each day to come from the stationary distributions of the models (we assume both the OU model and the VAR(1) model completely relax overnight).

An alternative to assuming the stationary distribution for the first measurement of the day, is to use the first measurement of each day only to condition upon for the second measurement, but ignore the first measurement in the evaluation of the min-log-likelihoods altogether. However, the models with measurement error require a chain of connected observations to optimize their predictive accuracy. For the models without measurement error, we nonetheless verified whether this decision has any significant impact on the results presented in this paper. This was not the case.

Some of the time series also include missing values. The time interval between two measurements with a missing value in between will in general be longer than the typical time interval between measurements not separated by a missing value. This may introduce a bias in favor of the continuous time OU model. Because we require a chain of consecutive observations for the models with measurement error, we included transitions between observations with missing values in between them. For the OU model, we then considered the actual (longer) time intervals between the measurements. For the VAR(1) model, we also considered a time interval that was twice as long than the other time intervals when there was a missing observation (i.e., $\Delta t = 2$ instead of $\Delta t = 1$); if there were even more missing values in between observations, the time interval was adjusted accordingly. To verify whether this decision had any impact on the results, we also compared the predictive accuracy of the models without measurement error for time series for which such transitions involving missing values are not included; this did not significantly alter any of the results presented in the paper.

## Results

**Figure 2:**
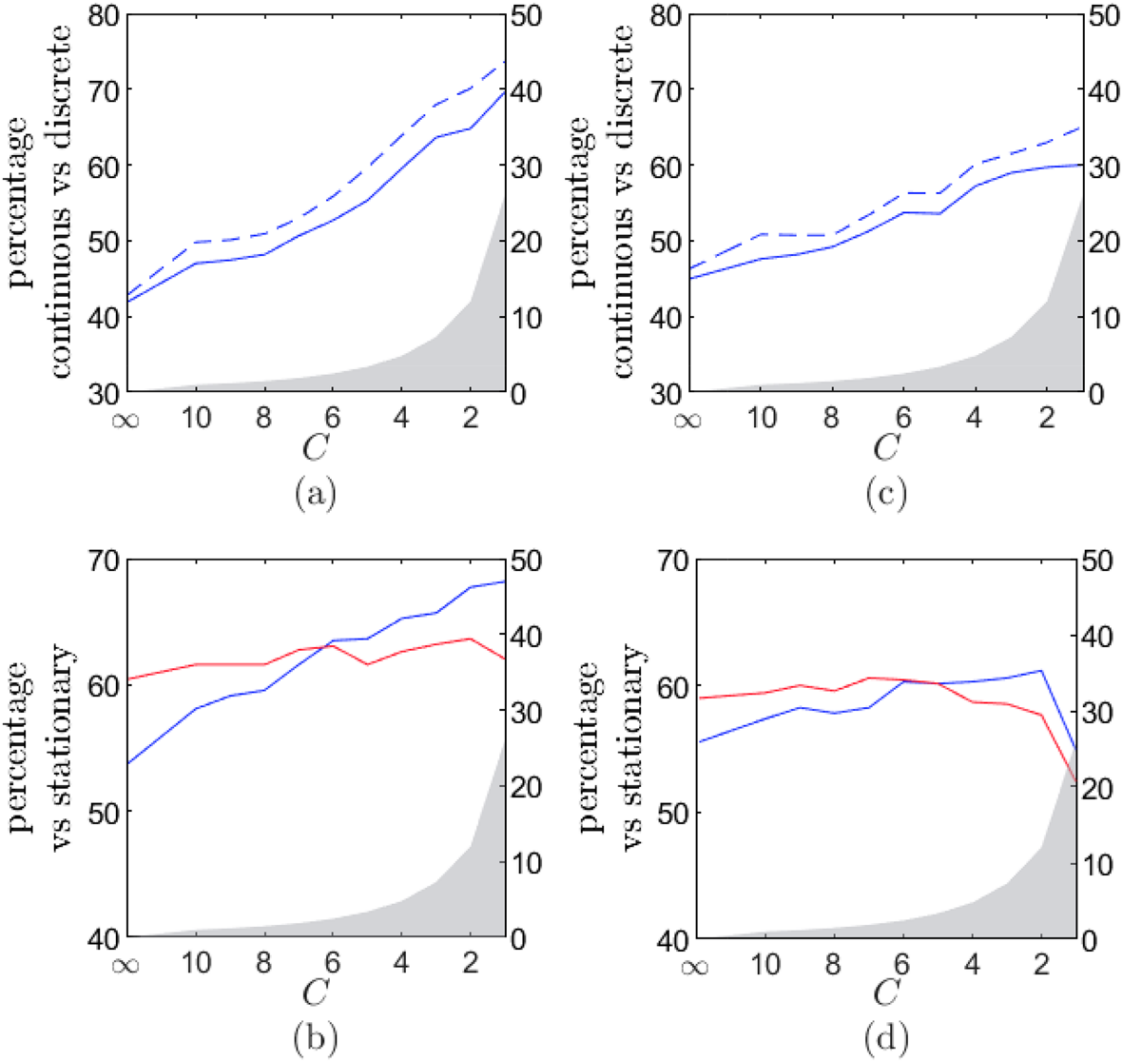
Comparison of the predictive accuracy of several models. In each graph, the *x*-axis refers to the cutoff criterion for large deviations. From left to right, more observations are removed.The grey shaded area depicts the percentage of data points that are on average removed in function of the critical value (see rightmost *y*-axis). The critical value $C = \infty$ corresponds to the case where no data are removed. The *y*-axis refers to the model comparison. (**a**) Comparison of the basic OU vs the basic VAR(1) model. The solid line shows the percentage of time series for which the basic OU model has a better predictive accuracy than the basic VAR(1) model as a function of the critical value *C* for removing large deviating speeds. The dashed line shows the same, but is based on a subset of time series for which either the VAR(1) or OU model outperformed its stationary variant (or both). (**b**) The percentage of time series for which the OU outperforms its stationary distribution (blue line) and the same for the VAR(1) model (red line). Panels (**c**) and (**d**) are equivalent to panels (**a**) and (**b**), except that they contain the results pertaining to the models with measurement error.

An overview of the results is provided in Fig. 2. In Panel (a), the percentage of cases for which the OU model has a better predictive accuracy than the VAR(1) model is depicted in function of the critical value *C*. From left to right, an increasing number of data points is removed (as indicated by the grey shaded area). For the data with all observations included ($C = \infty$), the OU model is slightly outperformed by the VAR(1) model for the majority of the time series (the OU model wins from the VAR(1) model for only 42% of the time series). For these data, the predictive accuracy does not improve for the majority of the time series when time intervals are explicitly taken into account, which is contrary to what we expected.

As the solid line in Panel (a) shows, decreasing the value of *C* (and therefore removing more data points), leads to an increase of the predictive accuracy of the OU model. Initially, when the criterion is still very strict, for example with $C = 10$, only a very small number of data points are removed, yet a remarkable improvement in the predictive accuracy of the OU model in comparison to VAR(1) model can be seen. It seems as if sometimes removing a single data point can be very beneficial for improving the predictive accuracy of the OU model compared to the VAR model. Lowering *C* further, the OU model starts outperforming the VAR(1) model in almost 70% of the cases.

Panel (b) depicts the predictive accuracy of the OU model compared to its stationary distribution (in blue) and the same for the VAR model (in red). For all data ($C=\infty$), the OU model outperforms a simple stationary model in but 54% of the cases, while the VAR(1) model outperforms a stationary model in 60% of the cases. If the reason for the stationary model having a better predictive accuracy were that there is almost no autocorrelation in the data, we would expect the OU and the VAR(1) model to be outperformed by the stationary model an equal number of times. Yet, the large difference in the percentages indicates that there is something else going on for several of the time series. As the large deviations removal criterion becomes less strict and more data points are removed, the predictive accuracy of the OU model steadily keeps on improving in comparison to that of a stationary model. However, this is not the case for the VAR model and around $C = 6$, the OU model is better as a dynamical model (in comparison to the stationary model) for more time series than the VAR model. It can be seen in Panel (a) that this coincides with the point at which the OU model overtakes the VAR model in terms of predictive accuracy. From $C=6$ onward in Panel (a), the OU model has predictive accuracy for the majority of the time series in our data. The fact that the VAR(1) model does not benefit from removing large deviations is an indication that large changes on small time scales indeed obscure the dynamics for the OU model, but that this effect is less present for the VAR(1) model.

Maybe the fact that for a substantial minority of the time series the stationary model wins from the dynamical counterpart biases the results in Panel (a). Therefore, we have repeated the analysis with the subset of time series for which the dynamical model wins. The dashed blue line in panel (a) depicts the percentage of time series for which the OU model outperforms the VAR(1) model in terms of predictive accuracy when those time series for which a stationary model has got the best predictive accuracy are neglected. For those time series, neither the OU model nor the VAR model can properly identify meaningful dynamics (meaningful for prediction).

Panels (c) and (d) of Fig. 2 have a similar structure as Panels (a) and (b) but are for the models with measurement error. The OU model benefits slightly more from being complemented with measurement error than the VAR(1) model. The OU model with measurement error has a better predictive accuracy than its error-free counterpart for 56% of the time series, while the VAR(1) model with measurement error has a better predictive accuracy in 53% of the cases. Nevertheless, in panels (c) and (d) we see a very similar pattern as in panels (a) and (b). Without correcting for large deviations, adding measurement error does not lead to a better predictive accuracy for the OU model when compared to the VAR(1) model. We do again see significant improvements when large deviations are removed, even when only a small number of data points are considered as large deviations. This suggests that the large fluctuations on small time intervals are probably not (solely) caused by measurement error. Therefore, it seems that other dynamics are at play which introduces dynamical patterns in the time series which diverge from the relaxation patterns expected in an OU model.

In contrast to panel (b), we can see in panel (d) that removing too many outliers will ultimately harm the predictive accuracy of the models. It is likely that when too many data points are removed, the parameters become poorly estimated on the training data that are left (this was less a problem for the simpler models). This has ramifications for the predictive accuracy.

The time series on which Fig. 2 is based are constructed by aggregating the positive and the negative affect items into PA and NA constructs. Thus all models are bivariate PA-NA models. To rule out the possibility that the results only hold for the PA-NA constructs, we redid our analyses for the basic models without measurement noise on two raw affect items, that is, *happy* and *depressed*. The results of these analyses can be found in Appendix C. The qualitative results described in this section were retrieved for the raw items as well.

Although Akaike’s information criterion^22^ (AIC) does not properly take into account the difference in model complexity between the OU model and the VAR(1) model, we nonetheless also compared the two models using the AIC. An in detail discussion on the AIC and our results can be found in Appendix D. Here, we summarize the findings. For the raw data (not considering large deviations), the OU model outperforms the VAR(1) model  in 58% of the time series in terms of AIC. Although this is better than the 43% obtained for the walk-forward cross-validation analysis, we again see that the continuous time model profits significantly from removing small numbers of large deviations. When doing this, the OU model starts outperforming the VAR model in almost 80% of the cases.

## Conclusion

Starting from the idea that intra-individual processes in psychology are assumed to continuously unfolding across time, and that therefore time information should play an important role when trying to model such processes, we set out to investigate how much the predictive accuracy of discrete-time vector autoregressive (VAR) models can be improved by recasting them in a continuous-time framework. We showed that a one-to-one correspondence exists between the continuous-time Ornstein–Uhlenbeck (OU) process and the VAR(1) model when the process has been observed at regular time intervals. Yet, in practice, as is the case with ESM studies, time intervals are generally not of equal length and there are often benefits to choosing unequal, even randomized, lengths. However, VAR(1) models are more frequently used as an analysis tool than the continuous-time OU model, even though the processes being studied are commonly thought of as evolving continuously.

It had already been shown that the VAR(1) model can lead to severe biases when it is used to fit time series that have been generated using a continuous-time OU model with unequally spaced time intervals. These results suggest that care has to be taken when interpreting results obtained with a discrete-time model when analyzing continuous processes on unequally spaced time intervals. By extension, one would assume that in general better predictions can be made when time intervals are explicitly taken into account. The continuous-time model explicitly takes into account the elapsed time since the previous observation to make a prediction about the current state of the system, thus accounting for the fact that uncertainty about the current state grows with time.

Nevertheless, when out-of-sample cross-validation is used to assess the model’s predictive accuracy on typical ESM data in affect research, the discrete-time VAR(1) model outperforms the continuous-time OU model in the majority of the cases, even though the measurements of the underlying the time series are unequally spaced. To better understand this counterintuitive result, we also compared the predictive accuracy of these two dynamical models to a stationary model, which is simply a Gaussian distribution (conditional and marginal distributions in the VAR(1) model and OU model are always Gaussian). The results showed that there are many occasions in which a stationary model has a better predictive accuracy, especially when compared to the continuous-time model. This could suggest that there are many time series in which there are no apparent dynamical patterns. However, if that were the case, we would expect the stationary model to outperform both the VAR(1) and OU model an equal number of times, more or less. The asymmetry in the results therefore hints in a different direction.

In the paper, we briefly described the specific relaxation dynamics encompassed by the OU model and we argued that deviations from these dynamical patterns, even if they occur only occasionally, can harm the predictive accuracy of the OU model. When trying to fit the model, they introduce certain trade-offs. Especially large changes on short time intervals may lead to an underestimation of the relaxation time, making it seem as if no dynamical patterns are present (as if there is no autocorrelation), although the time series might nicely follow the expected dynamical patterns except for these abrupt changes. In such a situation, it is possible that a stationary model also works better, because the dynamical model is unable to account for the dynamical patterns that are present in the data. Large inexplicable changes (changes with a magnitude that is larger than the typical change observed in the time series) can also be harmful for a VAR model, but since the dynamics of the OU model and the VAR(1) model differ slightly when time intervals are unequally spaced, they do not have to affect both models in the same way. In practice, divergent dynamics can be a consequence of measurement error, which naturally augments random fluctuations in the observed process. They could also be caused by external events impacting the dynamics of the system. We consider both of these possibilities in our analyses.

To account for measurement error, we complemented the OU model and the VAR(1) model with a measurement error process and repeated the analyses. Although the continuous-time model benefits more from adding measurement error, the discrete-time model still has a better predictive performance. This suggests that measurement error might not be the underlying cause for the apparent discrete behavior. When we start ignoring extreme (outlying) changes on short time intervals in model estimation, however, we see that the continuous-time models start outperforming the discrete-time models. We then also notice that the continuous-time models increasingly more outperform the stationary model, and that this is not the case for the VAR(1) model. Already when only a very small number of abrupt changes are removed do we see a major improvement for continuous-time models. As more data get removed, the continuous-time models steadily improve (not only in terms of predictive accuracy but also in terms of pure fit).

These results indicate that there are indeed continuous dynamical patterns present in ESM data. However, these are not the only effects that are present. There is evidence for abrupt and large changes that are inexplicable from the perspective of an OU model. If not properly accounted for, these abrupt changes obscure the continuity of the dynamics resulting in bad model estimates and low predictive accuracy. The nature of these abrupt changes is currently unknown. In this paper, we focused on affect time series and it is conceivable that these changes were caused by events in the lives of the participants which strongly influenced and disrupted their natural affect system. Another explanation could be that the dynamics underlying affect, although continuous, are governed by a different model than the OU model, resulting in misfit. It could be, for instance, that the assumption of Gaussian random fluctuations is not adequate for describing affect dynamics. An alternative possibility is that the inexplicable deviations are caused by non-Gaussian measurement error, something which is not properly accounted for by a Gaussian measurement error model.

Our work has its limitations. First, our results are valid for the sample of several hundred participants whose time series have typical lengths as often encountered in ESM studies. Hence, we believe it is justified to generalize this result to ESM data of this kind. However, subsequent studies should be carried out to investigate whether the conclusion also holds for longer time series or for studies with a different sampling scheme (e.g., longer time intervals between measurements). Second, we refrain from drawing conclusions regarding the ‘true’ model underlying affect dynamics. In order to do this, additional work would be required. For example, a deeper understanding is needed about the large deviations. We can make suggestions for possible causes (as we did above), but the true nature of these deviations requires additional empirical work.

In conclusion, we have compared various models (discrete-time and continuous-time, with and without measurement error) for a large number of typical ESM time series. For the data considered in this paper, the continuous-time models do not outperform the discrete-time variants as one would naively assume. However, removing a fraction of large deviations does tip the balance in favor of the continuous-time model and thus gives evidence for a underlying process unfolding in continuous time. An agenda for future research is the identification and explanation of the large deviations—are these non-Gaussian measurement errors or do they signify specific contextual influences?

## Supplementary Information

Supplementary information.

## Data Availability

The data that was used for the analyses presented in this paper can be made available upon request.

## Code availability

The code that was used to obtain the results in this paper can be shared upon request.
