# Supplementary material for: A comparison of continuous and discrete time modeling of affective processes in terms of predictive accuracy
Source: Sci Rep. 2021 Mar 18;11:6218. doi: 10.1038/s41598-021-85320-4 (PMC7973711; doi:10.1038/s41598-021-85320-4)
Supplement: Supplementary file 1 — Supplementary information. [file 41598_2021_85320_MOESM1_ESM.pdf]

A comparison of continuous and discrete time modeling of affective processes in terms of predictive accuracy

Tim Loossens[1*], **Francis Tuerlinckx**[1], **and Stijn Verdonck**[1]

[*]tim.loossens@kuleuven.be
[1]KU Leuven, University of Leuven

A The Ornstein-Uhlenbeck model

The integral formulation of the OU process is[12]

$$\mathbf{y}(t_i+\Delta t_i) = \left(I_d - e^{-\theta\Delta t_i}\right)\mu + e^{-\theta\Delta t_i}\mathbf{y}(t_{i-1}) + \int_0^{\Delta t_i} e^{-\theta(\Delta t_i - t')}\sigma\,\mathrm{d}\mathbf{W}(t'). \tag{A.1}$$

When the time intervals between the measurement occasions are equidistant with duration Δt we have,

$$\mathbf{x}_i = \mathbf{y}(t_{i-1}+\Delta t).$$

In terms of the measurements $\mathbf{x}_i$, expression (A.1) then becomes

$$\mathbf{x}_i = \left(I_d - e^{-\theta\Delta t}\right)\mu + e^{-\theta\Delta t}\mathbf{x}_{i-1} + \int_0^{\Delta t} e^{-\theta(\Delta t - t')}\sigma\mathrm{d}\,\mathbf{W}(t').$$

This expression has the form of a VAR(1) model with

$$\mathbf{c} = \left(I_d - e^{-\theta\Delta t}\right)\mu \tag{A.2}$$

$$A = e^{-\theta\Delta t} \tag{A.3}$$

$$\varepsilon_i = \int_0^{\Delta t} e^{-\theta(\Delta t - t')}\sigma\,\mathrm{d}\mathbf{W}(t'). \tag{A.4}$$

The equations (A.2) and (A.3) indeed satisfy the properties of the stationary mean of the VAR(1) model. It can be shown that the innovations in expression (A.4) also satisfy the properties of the innovations of a VAR(1) model: they are Gaussian distributed with mean zero and they are uncorrelated across time.

Just like the connection (3) between the covariance Σ_ε of the innovations and the covariance Σ of the measurements, there is a link between the covariance $\sigma\sigma^T$ of the Wiener processes in equation (6) and the covariance $\Sigma_\mathbf{y}$ of the stationary distribution of $\mathbf{y}$ (provided it is stable):

$$\theta\Sigma_\mathbf{y} + \Sigma_\mathbf{y}\theta^T = 2\sigma\sigma^T. \tag{A.5}$$

This Sylvester equation specifies what the covariance $\Sigma_\mathbf{y}$ will be, given the system interactions θ and the covariance $\sigma\sigma^T$ of the Wiener processes determining the stochastic fluctuations on an infinitesimal timescale. By virtue of the relation (5), we expect the stationary (time-independent) distribution of $\mathbf{y}$ to have the same stable mean μ and the same covariance Σ as the measurements $\mathbf{x}_i$ (which also follows from the equivalence (A.2-A.4)).

B Verification of the min-log-likelihood procedure

In the paper, we use maximum likelihood optimization to obtain the parameter estimates of both the OU model and the VAR(1) model. In practice, we minimize the min-log-likelihood and we use the differential evolution global optimization heuristic to do this. There exists a closed-form expression for the parameter estimates of the VAR(1) model, but we want to constrain the solutions to the VAR(1) model to those that can be derived from or mapped onto a stable OU process (measured at regular time intervals). These constraints can easily be imposed using the min-log-likelihood formalism. To fit a VAR(1) model, we essentially fit an OU model while assuming time intervals of equal length. Then, we transform the obtained OU estimates into their corresponding VAR(1) estimates.

To test our minimization procedure, we compared the VAR(1) estimates obtained from our method with their closed-form solution (provided that the closed-form solution was indeed associated to an OU model). In Figure B.1, the results of this analysis are depicted. The abscissas depict the closed-form least-squares (ls) estimates and the ordinates depict the min-log-likelihood (mll) estimates. When the estimates from both formalisms are equal, the dots fall on the main diagonal. Except for some few cases where the differential evolution has not yet fully converged, we see that both formalisms result in the same estimates.

C Analysis of two raw affect items

To rule out that the results of the paper are mainly a consequence of the fact that we aggregated all positive and negative items into PA and NA construct by respectively averaging them, we also did our analyses on two raw affect items, *happy* and *depressed*. We only repeated the analyses for the basic models without measurement error. In Figure C.1, we already see however that the qualitative results discussed in the paper also present themselves at the level of the raw affect items.

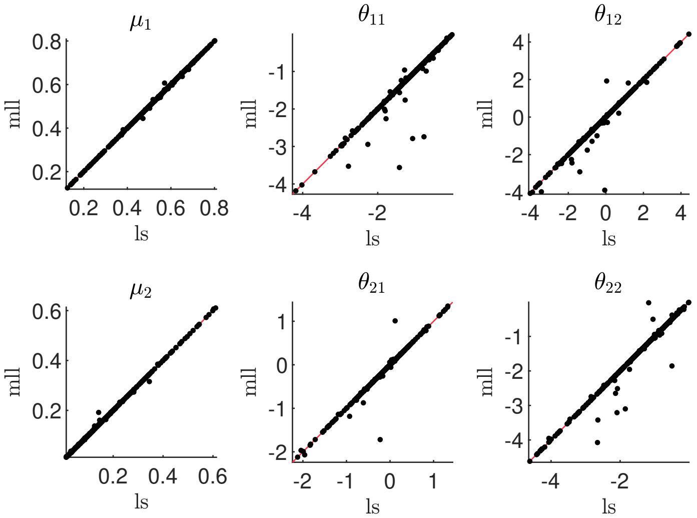

Figure B.1. Comparison of the VAR(1) estimates obtained using the min-log-likelihood optimization procedure (Differential Evolution) with those obtained using the least-squares formalism (traditional regression). Each panel corresponds to a different OU parameter (μ_1, μ_2, θ_{11}, θ_{12}, θ_{21}, θ_{22}). The abscissas depict the least-squares (ls) estimates and the ordinates depict the min-log-likelihood (mll) estimates. When both estimates are identical, the marker lies on the main diagonal. 508 estimates are included in this figure.

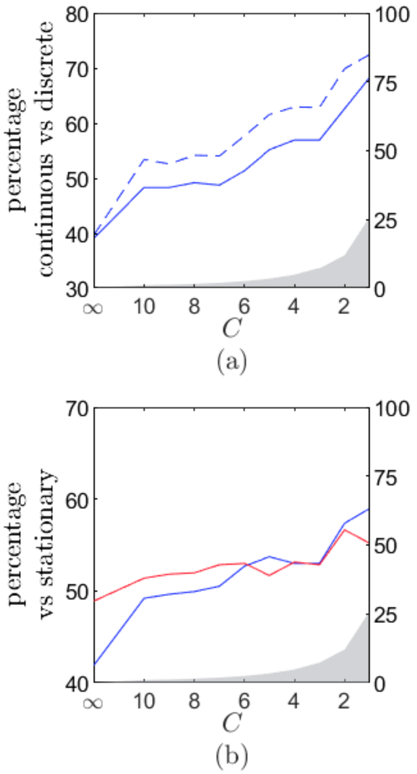

Figure C.1. Comparison of predictive accuracies. (a) The percentage of time series for which the OU model has a better predictive accuracy than the VAR(1) model as a function of the critical value C for removing large speeds. The dashed line shows the same, but only includes time series for which either the VAR(1) model or OU model outperforms the stationary model. The critical value $C = \infty$ corresponds to the case where no outliers are removed (i.e., the original data). The grey shaded area depicts the percentage of data points that are on average removed in function of the critical value. This percentage is indicated on the y-axis on the right. (b) The percentage of time series for which the a simple stationary distribution is outperformed by the OU model (blue) and the VAR(1) model (red).

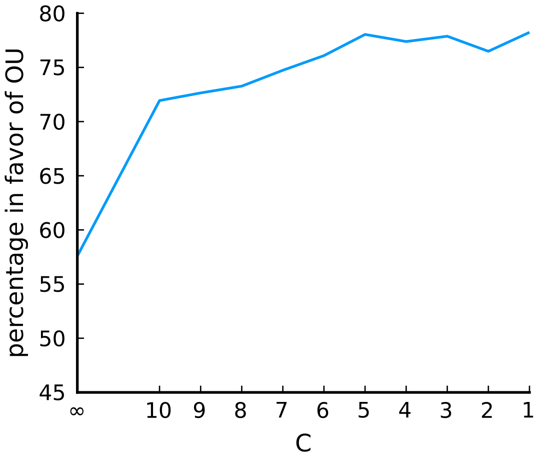

Figure D.1. Results for the AIC. The line depict the proportion of time series for which the continuous-time OU model has a better fit than the discrete-time VAR(1) model in function of the MAD cutoff C. When $C = \infty$, no data points are removed.

D Analysis using Akaike's information criterion

In the main text, we use out-of-sample prediction to compare the performance of the discrete-time VAR(1) model to that of the continuous-time OU model. The advantage of out-sample-prediction is that we can naturally penalize the models if they were to be overly complex, causing them to overfit.

Here, we use Akaike's information criterion (AIC) for model selection to compare the models[21]. In AIC is defined as

$$\mathrm{AIC} = 2k - 2\ln(L)$$

where k represents the number of free model parameters and L denotes the likelihood. The smaller the AIC, the better the model's performance. Since the AIC scales with the numbers of free parameters, models with a lot of parameters are penalized more severely by the AIC than models with less parameters. As such, the AIC does not only account for the fit of a model (through the likelihood), but also considers its complexity.

Since the VAR(1) and the OU model have the same number of parameters, comparing the AIC of the models for a given time series actually comes down to comparing their goodness-of-fit through their the min-log-likeloods:

$$\mathrm{AIC}_{\mathrm{OU}} - \mathrm{AIC}_{\mathrm{VAR}} = -2\left(\ln(L_{\mathrm{OU}}) - \ln(L_{\mathrm{VAR}})\right).$$

In other words, the model with the better goodness-of-fit (the better likelihood) will also be the model with the smallest AIC.

Because the VAR(1) model and the OU model treat time differently, they have different degrees of freedom regarding their time evolution (see the section on how VAR and OU deal with time). This is not taken into account by the AIC criterion. That is why we focus on out-of-sample prediction in the main text. Yet, here we compare the actual fit of the OU model with that of the VAR.

We consider the same time series as considered in the main text. Just like for the actual analyses, we consider several MAD cutoffs C for the detection of large deviations and we examine how the model comparison is affected by their removal. The results of this analysis are depicted in Figure D.1. In contrast the the out-of-sample analysis discussed in the main text, the OU model is not outperformed by the VAR(1) model for the majority of the time series when goodness-of-fit is considered. Instead, the OU model outperforms the VAR model for 58% of the time series when no data points are removed. Nevertheless, the same qualitative results can be observed as for out-of-sample predictions when deviations are considered. Already by removing only a very small proportion of the data ($C = 10$), we see a significant increase in the goodness-of-fit of the OU model compared to that of the VAR model. When less stringent cutoff values are considered, the OU model starts to outperform the VAR model in almost 80% of the cases.
